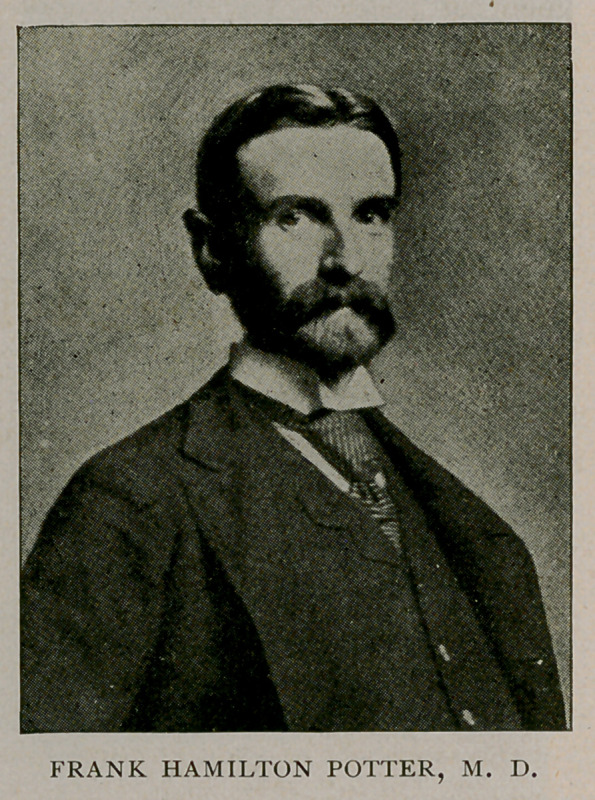# A Century of Medical History in the County of Erie.—1800-1900

**Published:** 1898-12

**Authors:** William Warren Potter

**Affiliations:** Buffalo, N. Y.


### A CENTURY OF MEDICAL HISTORY IN THE COUNTY OF ERIE.—1800–1900.

By WILLIAM WARREN POTTER, M. D., Buffalo, N. Y.

*Pioneer Physicians—Medical Societies—Medical Colleges—Hospitals—Medical Journals—Medical Officers of the Civil War—Women Physicians—History of Homeopathy—Individual Members of the Profession.*

[*Continued from the November edition.*]

### Providence Retreat.

THE Providence Retreat is a private institution for the care and treatment of the insane, conducted by the Sisters of Charity. It was opened July 15, 1861, on North Main street, near Humboldt Parkway, and now has capacity for 175 patients. Its grounds are ample and its lawns beautiful and well kept. Dr. William Ring was the first attending physician, and the medical staff is now made up as follows: Physician in charge, Harry A. Wood; assistant physician, John J. Twohey; consulting physicians, Conrad Diehl, Thomas Lothrop, E. C. W. O'Brien, James W. Putnam, Ernest Wende; consulting surgeon, Herman Mynter; consulting gynecologist, Henry D. Ingraham; consulting oculist, Alvin A. Hubbell.

### Erie County Hospital.

The law regarding state care of the insane that took effect in 1893 left vacant the commodious and substantial structure that had been used by the county as an insane hospital. Recognising the desirability as well as the economy of using this building as a hospital for the county sick, a number of physicians under the leadership of Dr. John H. Pryor, of Buffalo, brought this subject to the notice of the board of supervisors. After considerable debate and delay the Erie County Hospital was finally established and a visiting and consulting staff appointed. It was organised January 1, 1894, and is situated on North Main street, near the city line. The capacity

PROVIDENCE RETREAT.

of the hospital is about 400 beds and its average population, 350 patients. A consumption hospital annex has been constructed with a capacity for eighty patients. This building is separated from the main structure, and the theory that consumption is an infectious disease pervades the entire principles of its conduct.

The Erie County Hospital has a training school for nurses, of which Miss Sarah Bond Lowe was the first superintendent. She was assisted by five graduate nurses, each ward being placed under the supervision of one of these. The hospital staff at the present writing is made up as follows: Consulting physicians, Charles G. Stockton, A. T. Bull; consulting surgeons, Roswell Park, H. C. Frost, Marcel Hartwig, Wm. C. Phelps; consulting genito-urinary surgeon, De Villo W. Harrington; consulting gynecologists, Mathew D. Mann, Geo. T. Moseley; attending physicians, H. C. Buswell, De Lancey Rochester, Geo. A. Himmelsbach, Harry A. Wood, C. S. Jewett,

ERIE COUNTY HOSPITAL.

Truman J. Martin; attending surgeons, Edward J. Meyer, Eugene A. Smith, Herbert Mickle, John Parmenter; attending gynecologists,

CONSUMPTION ANNEX—ERIE COUNTY HOSPITAL.

Dewitt H. Wilcox, H. D. Ingraham, M. A. Crockett; attending obstetricians, E. L. Frost, Lawrence J. Hanley; attending ophthalmologists, A. A. Hubbell, Elmer Starr, A. S. Bennett, F. Park

Lewis; attending laryngologists, W. Scott Renner, H. J. Mulford; attending dermatologists, Grover W. Wende, Alfred Diehl; attending neurologists, Floyd S. Crego, James W. Putnam, Wm. C. Krauss; orthopedic surgeon, Bernard Bartow; genito-urinary surgeons, Byron H. Daggett, W. D. Greene, W. H. Heath; pediatrists, Maud J. Frye, W. E. Robbins, of Hamburg, N. Y.; attending pathologists, Herbert U. Williams, Earl P. Lothrop; assistants, A. E. Woehnert, in medicine; J. A. Gibson, in nervous diseases; Nelson Russell, in pathology; Jacob Meyer, in surgery; Herman C. Matzinger, in medicine; Wm. More Decker, in medicine.

### Buffalo Woman's Hospital.

This hospital was established by Dr. Thomas Lothrop in May, 1886, to receive and care for women, married or single, during childbirth, or while suffering from diseases peculiar to their sex. It was first located at the corner of Seventh and Maryland streets, but in May, 1891, it was removed to its present situation, 191 Georgia street, corner of Seventh, where it occupies a large and well-appointed building that has been remodeled to meet the requirements of such an institution. It receives a limited number of worthy indigent women suffering from curable diseases free of expense, provided they are known to be unable to pay for their board and treatment. There is also a free dispensary maintained in connection with the hospital. The private rooms are suitably furnished and supplied with all the comforts consistent with modern surgical cleanliness.

The pupils of Niagara University Medical College received their obstetric training in this hospital. Dr. Thomas Lothrop is physician-in-chief, and Dr. C. C. Frederick is surgeon-in-chief. The consulting staff attached to the service is composed as follows: Herman Mynter, R. L. Banta, H. C. Buswell, William Warren Potter, Herbert Mickle, Eugene A. Smith and Walter D. Greene.

### Saint Mary's Infant Asylum and Maternity Hospital.

This institution is located at 126 Edward street, Buffalo, near Delaware avenue, and is under the charge of the Sisters of Charity, the chief of whom is Sister Maria, Superior. Under the original charter there were two institutions. A widows' asylum was organised January 12, 1852, and St. Mary's Lying-in Hospital was chartered October 25, 1855. Two cottages were opened June 15, 1854, with accommodations for fifteen inmates. The buildings are now large and commodious structures with all modern improvements. The two institutions were consolidated October 18, 1897, under the official name given at the head of this section.

Dr. James P. White was the first attending physician with Sister Rosalie in charge, assisted by two other Sisters of Charity. After Dr. White's period of service terminated, Dr. James S. Smith took his place, and he in turn was followed by his son, Dr. Eugene A. Smith.

BUFFALO WOMAN'S HOSPITAL.

The two Doctors Smith still render medical service at the hospital, which has accommodations for nearly 200 patients.

Dr. Thomas Lothrop is consulting physician and Drs. H. D. Ingraham, C. C. Frederick, William K. O'Callahan and Earl P. Lothrop render further professional services when such are needed.

### St. Francis Asylum.

This institution was established December 18, 1861, and is located at 337 Pine street. The founder, Mother M. Francis Bachman with the Sisters of the Franciscan Order came from Philadelphia, where they had established a similar asylum. It has for its object the care of the aged poor of both sexes regardless of nationality or religious denomination. The average number of inmates from 1863 to 1867 was nineteen; during the past ten years the average has been 245. At present there are about 300 inmates in the institution and the number of sisters in attendance is thirty-two. The total number of Franciscan Sisters is 170, who are engaged in the various institutions of the order located throughout the country. Formerly Drs. Edward Storck and Conrad Diehl were attending physicians; now Drs. Thomas Lothrop, J. D. Flagg, William C. Krauss and A. E. Persons constitute the attending staff.

### Buffalo Children's Hospital.

This hospital was established in September, 1892, through the generosity of Mrs. Gibson T. Williams and Miss Martha T. Williams, who purchased the property at 219 Bryant street, and after refitting it, offered it rent free to the board of managers, which is composed of a group of philanthropic women. The hospital has accommodations for about fifty-two patients. The following is the present list of officers: President, Mrs. Lester Wheeler; first vice-president, Mrs. George H. Lewis; second vice-president, Mrs. William Hamlin; purveyors, Mrs. Henry Watson, Mrs. Bainbridge Folwell; treasurer, Miss Martha T. Williams; secretary, Mrs. Bernard Bartow; executive committee, Mrs. E. B. Alward, Mrs. George Truscott, Mrs. S. S. Spaulding, Mrs. Henry Bull, Mrs. Nathaniel Rochester, Mrs. John L. Williams, Mrs. Dexter P. Rumsey, Mrs. Charles Pardee, Mrs. Edwin Bell, Mrs. George Parkhurst, Mrs. Edmund P. Fish, Mrs. Joseph Hunsicker, Mrs. Charles B. Wheeler; advisory committee, Sherman S. Rogers, Henry W. Sprague, G. L. Williams, C. Sidney Shepard, Bernard Bartow, John Parmenter. Medical staff, Bernard Bartow, orthopedic surgeon; John Parmenter, attending surgeon; H. Y. Grant, ophthalmic and aural surgeon; Charles S. Jones, Dewitt H. Sherman, attending physicians; W. Scott Renner, laryngologist; Loren H. Staples, assistant surgeon; H. G. Matzinger, pathologist.

It has a training school for nurses, in which the course is two years, and there are nine nurses at present on duty. Miss Olivia Moore is the superintendent of the hospital.

GERMAN DEACONESS'S HOME AND HOSPITAL.

One of the latest hospitals to be organised in this city is the German Deaconess's Home and Hospital, situated on Kingsley street, near Humboldt Parkway. About four years ago the establishment of this hospital was suggested and a number of private meetings were held to consider its feasibility. After careful deliberation it was decided to call a public meeting, which was held February 26, 1895, in St. Paul's German U. E. Church, Ellicott street, at which plans were presented to the assemblage. The interest manifested was such as to justify the organisation of a society whose object is to further the interest of the work, and it is known as the Deaconess's Association of Buffalo. The association rented a building on Goodrich street, October 23, 1895, and the first patient was admitted November 14, 1895. In the spring of 1896 the erection of a new and commodious building was taken into consideration. Plans were adopted, a site secured, and the construction of the building was commenced. It was dedicated November 21, 1896, and is now in full operation.

THE GERMAN DEACONESS'S HOME.

The building consists of three distinct divisions: The central or main division is intended as a home for deaconesses and working women, the east wing will be used for hospital patients, and the west wing will be occupied as a home for aged men and women. Each division will accommodate forty inmates. The hospital is admirably arranged and consists of a basement, a kindergarten or crêche, a polyclinic room, apothecary's room, and an office. On the first floor are two wards for men and a small children's ward, five private rooms, a day room and a diet kitchen. On the second floor is an operating room connected with a medicine room and a preparing room. There are also two wards, five private rooms, a day room and a diet kitchen for women patients; on the third floor are eight rooms to be fitted up when occasion demands.

The success of the enterprise is due largely to the persistent efforts of the Rev. Carl Schild, who has been chosen president of the board of directors. The generosity of J. F. Schoelkopff, Esq., who gave $5,000, should be mentioned, while the medical department owes much to the activity and thoughtfulness of Dr. E. A. Smith.

The management of the house is under the general supervision of a sister superior, Miss Tobschall, known to the inmates as Sister Ida. Miss Mary Barth, a graduate nurse, has been engaged as superintendent of the hospital department, and Miss Eliza Loy is to have charge of the home for the aged. The medical staff of the hospital is made up as follows: Consulting physicians, Conrad Diehl, Louis Schade, Charles Wetzel; attending physicians, De Lancey Rochester, William Gärtner; attending surgeons, Herman Mynter, Roswell Park, E. A. Smith; gynecologist, M. D. Mann; ophthalmologist, Edmond Blaauw; laryngologist, W. Scott Renner; dermatologist, Alfred E. Diehl; diseases of children, Irving M. Snow; neurologists, James W. Putnam, William C. Krauss; pathologists, H. U. Williams, Earl P. Lothrop; obstetrician, H. G. Bentz; resident physician, Frederick A. Mendein.

### Riverside Hospital.

The Riverside Hospital of Buffalo was founded by Dr. Lillian Craig Randall in 1892. It was first opened at 2393 Niagara street, near the river bank, and at that time consisted of two rooms, containing three beds. Within a year the need for more commodious quarters became imperative and it was removed to Breckenridge street. After an interval of eighteen months it again became necessary to find more room and the institution was removed to its present location, 306-308 Lafayette avenue, where it now carries over thirty beds.

The primary object in view in starting the hospital and the feature which has contributed so largely to its success, was to provide an institution where patients could have hospital advantages and at the same time remain absolutely under the control of the family physician. The greater part of the work done is surgical, though all kinds of cases are taken, except contagious diseases and insane.

In connection with the hospital is a training school for nurses, the course covering two years and consisting of regular lectures in addition to their clinical instruction. Six nurses have been graduated, five of whom now hold superintendencies of hospitals. The following staff of well-known physicians is connected with the institution: Physician in charge, Lillian Craig Randall; attending physicians, John Parmenter, Henry D. Ingraham, John C. Thompson, Julius H. Potter, Vertner Kenerson; consultants—surgery, Roswell Park; medicine, Charles G. Stockton, De Lancey Rochester; diseases of the nose, ear and throat, Geo. F. Cott; diseases of the eye, Alvin A. Hubbell, Arthur G. Bennett, Elmer E. Starr; neurologist, William C. Krauss; dermatologist, Grover W. Wende;

THE RIVERSIDE HOSPITAL.

orthopedic surgeons, Bernard Bartow, E. C. W. O'Brien; pathologist, H. G. Matzinger; house surgeon, Thomas McKee; house physician, Carro Julia Cummings.

### City Hospital for Women.

A hospital with the above name was established in 1896 by Dr. Charles E. Congdon. It is located at 859 Humboldt Parkway and receives women for care, operation and treatment who are suffering from diseases pertaining to their sex. The medical staff is composed as follows: attending physicians, Charles E. Congdon, James S. Porter, John J. Walsh, C. B. LeVan and A. L. Benedict; consulting physicians, F. E. Harrington, Frederick Preiss, Thomas F. Dwyer, Emil Lustig, Julius F. Krug, W. C. Callanan and William G. Ring; obstetrics and gynecology, William Warren Potter and Henry D. Ingraham; general surgery, Edward M. Dooley and Eugene A. Smith; ophthalmology and otology, A. A. Hubbell and J. J. Finerty; nervous diseases, Floyd S. Crego; bacteriologist, Frank J. Thornbury. Dr. Congdon is the surgeon in charge and to his energy is largely due the success of the hospital.

### German Hospital and Dispensary.

This hospital and dispensary was organised in 1895. The dispensary was opened December 14, 1896, at 621 East Genesee street, Buffalo, with the following-named physicians as members of the staff: President, Charles H. W. Auel; vice-president, Gustav Pohl; secretary Max Breuer; house committee, L. Schroeter, Sigmund Goldberg and Henry G. Bentz; general medicine, E. E. Koehler, Fridolin Thoma, Julius Ullman; surgery, J. G. Meidenbauer and M. Hartwig; consulting surgeon, Herman Mynter; diseases of women, C. H. W. Auel, Max Breuer and Sigmund Goldberg. Psychiatry and diseases of the nerves, William C. Krauss, H. G. Matzinger and William Meisberger; diseases of children, L. Schroeter, Gustave Pohl, C. H. W. Auel; ophthalmology and otology, attendant, E. Blaauw; consultant, Lucien Howe; genito-urinary and skin diseases, J. M. Kraus, A. Jokle and G. W. Wende, with Ernest Wende as consultant.

The aim of this dispensary is to accept none but patients absolutely too poor to pay, and the list is scrutinised each week by a committee of three directors, with a view to ascertain if any patients not entitled to charity are receiving treatment. This committee, as well as every attending physician, is expected to become familiar with the social condition of each patient and to reject the applications of such as are found able to pay for medical treatment.

### IV.—Medical Journals.

### The Buffalo Medical Journal.

The desirability of establishing a medical journal in Buffalo had been agitated for some time previously, but definite plans were not made until the spring of 1845. A guaranty was signed by Alden S. Sprague, Austin Flint, Frank Hastings Hamilton and James P. White, protecting the publishers against loss, which resulted in the consummation of plans that had been languishing. It is proper to state that the guarantors were never called upon for funds, as the JOURNAL was self-supporting from the start.

In June, 1845, the first number of the BUFFALO MEDICAL JOURNAL was published under the editorship of Dr. Austin Flint, who was

BUFFALO

MEDICAL JOURNAL

AND

MONTHLY REVIEW

OF

MEDICAL AND SURGICAL SCIENCE.

EDITED BY AUSTIN FLINT, M. D.

**Vol. I.—JUNE.—No. I.**

BUFFALO:

PUBLISHED BY JEWETT, THOMAS & CO.

Office of Commercial Advertiser.

...............

1845.

*Facsimile of Front Cover Page—One-third size.*

its founder and owner. It was printed by Jewett, Thomas & Co., at the office of the *Commercial Advertiser* and consisted of twenty standard octavo pages. It contained an introductory editorial by Dr. Flint that occupied two and one-fourth pages; notes of a European trip, by Frank H. Hamilton, then professor of surgery at the

Geneva Medical College; cases of acute rheumatism treated by nitrate of potash in large doses, by Alden S. Sprague; a case of aortitis, with autopsy and remarks, by George N. Burwell; a case of hydrophobia, reported by James P. White; a case of midwifery with twins at different stages of development, by H. N. Loomis, and the last four pages of this number were filled with paragraphs under the general head, editorial, medical intelligence, bibliographical notices and miscellany.

At that time Buffalo contained less than 30,000 inhabitants, and though there were about seventy physicians of all sorts and conditions, one-half of whom were regulars, there were yet no medical societies organised in the city. The JOURNAL, however, was a success from the start owing to the energy of its editor and his associates and the united support of the regular medical profession. The first volume contained a total of 284 pages, but the second grew to an aggregate of 758 pages, which was the standard it maintained for many years.

Mr. James N. Matthews, afterward editor and proprietor of the *Buffalo Morning Express*, worked as a compositor on the first numbers of the JOURNAL and he stated to the writer in a conversation on the subject a short time before his death, that at first he experienced great difficulty in deciphering Dr. Flint's copy, as he prepared it for the press something after the manner of Horace Greeley.

The history of the BUFFALO MEDICAL JOURNAL involves the history of the medical profession of Buffalo for more than fifty years. In its pages are recorded the principal medical events that have occurred here during the half century of its existence, some of which are somewhat startling in character, while many are given in detail. It contains the reports of clinical cases and medical and surgical items that served to make the men of the early days famous.

In its fourth number is published the first information concerning the true nature of the infection of typhoid fever, as noticed on a previous page. A well in North Boston, Erie County, became poisoned by the excreta of a typhoid patient brought from Massachusetts. Twenty-one cases occurred in five families, all living within a few rods of the fatal well and deriving their water supply from that source. After seven had died Dr. Flint visited the locality, diagnosticated and traced the disease, then unknown in this region, from New England to North Boston, definitely establishing its contagion and pointing out its source. The published report became a classic in medical literature and formed the basis of a series of essays published in the

BUFFALO MEDICAL JOURNAL. It was Dr. Flint's first conspicuous success, and it is more than probable that it laid the foundation of his future fame as a clinician.

Dr. Frank H. Hamilton published in the pages of the JOURNAL his surgical clinics and fracture tables, together with other papers

JAMES N. MATTHEWS.

(*Of Matthews, Northrup & Co.*)

First Compositor of the Buffalo Medical Journal.

that served to form the basis of his future classic treatise on fractures and dislocations, that is recognised in every country throughout the civilized world, and has been translated into several tongues.

Dr. James P. White lent his powerful influence in support of the JOURNAL from the first, and published in its columns essays and clinical reports from which sprang a fame that made him known in two hemispheres. Every new method of procedure or new-fashioned instrument that came to his knowledge was made known to his colleagues through the JOURNAL.

Dr. Flint conducted the JOURNAL as sole editor fiom 1845 until 1853, when, having been invited to teach the piactice of medicine at Louisville, Ky., he transferred it to other hands. Meanwhile a young man from Mendon, N. Y., had been contributing a series of articles to its columns, under the name of "Smelfungus," that had attracted great attention on account of their rare wit, wisdom and originality. This young man was invited to become demonstrator of anatomy at the Buffalo Medical College, which circumstance made it convenient for him to transfer his residence to Buffalo. Recognising his talent and fitness for the work, Dr. Flint made haste to invite "Smelfungus" to become associated with him in the editorial conduct of the JOURNAL. Thus Dr. Sanford B. Hunt, "Smelfungus" no longer, without experience in journalism, indeed with very little experience of any kind, became practically editor-in-chief with the issue of July, 1853. The wisdom of this selection was never challenged, and two years later Dr. Flint conveyed his entire interest in the JOURNAL to Dr. Hunt, so in June, 1855, the latter became its sole editor and proprietor.

JULIUS F. MINER, M D.

During Dr. Hunt's administration, from 1853 to 1858, the JOURNAL enjoyed the most brilliant period in its history. Putting his whole talent and energy into the work, the editor soon made his journal famous, not only for the sparkling originality of its editorial department, but also for its journalistic *esprit de corps*. Dr. Hunt was a ready writer, an original thinker, and had special aptitude for editorial work. His ideas ran faster than his pen; hence it was difficult for him to keep his thoughts in check while his pen caught up to his expressions.

In the year 1855 the JOURNAL had its first experience as a defendant in a libel suit. The circumstances leading up to this event may be thus briefly stated: Dr. John D. Hill had been expelled from the medical society of the county of Erie for a violation of its rules and the JOURNAL had seen fit to make fearless and independent comment thereon. Fancying himself injured thereby Dr. Hill brought suit for libel against the editors, Drs. Flint and Hunt. The JOURNAL was mulcted in $500 damages by a jury that the editor, from his comments at the time, evidently thought below the average intelligence. It is proper to state in this relation that Dr. Hill was subsequently restored to membership by a mandate of the court, and was elected president of the society in 1887, as is recorded elsewhere.

A. R. DAVIDSON, M. D.

In addition to his duties as editor, Dr. Hunt was professor of anatomy in Buffalo Medical College and city editor of the *Commercial Advertiser*. Finally he became editor-in-chief of the *Commercial*, surrendered his professorship in 1858, and also transmitted his interest in the JOURNAL to other hands. A little later he was elected superintendent of public schools, and when the civil war came he joined the army as surgeon of United States Volunteers. He was placed in charge of Convalescent Camp, near Alexandria, Va., in 1863, a duty which enabled him to exercise his talent as an organiser. After the war he edited a volume known as the History of the United States Sanitary Commission, and upon completion of this task he became editor of the Newark (N. J.) *Daily Advertiser*. Later he published the Sunday edition of that newspaper, which is now conducted by his son, Mr. William T. Hunt. In January, 1884, Dr. Hunt was seized with a fatal illness, of which he died at Irvington,

N. J., April 6, 1884. His ashes repose in Forest Lawn Cemetery at Buffalo.

From 1858 to 1860 Dr. Austin Flint, Jr., was editor, but the proprietorship had been transferred to Mr. A. I. Mathews, then a well-known druggist in Buffalo. Now came a period of disaster. The prosperity that throughout had attended the JOURNAL seemed near its end. Mr. Mathews prostituted its advertising columns to the printing of quack advertisements. Thereupon the profession withdrew its support. Dr. Flint resisted the action of the druggist with all his might, but he was unable to stem the tide and the JOURNAL ceased publication for a time.

F. R. CAMPBELL, M. D.

Plans soon began to be discussed among leading physicians looking to its resuscitation, but as Mr. Mathews owned a proprietorship in the name of the JOURNAL it became inexpedient for a time to revive it. Finally, however, this difficulty was overcome, and in August, 1861, the JOURNAL was reëstablished under the able editorship of Dr. Julius F. Miner, the well-known surgeon. A slight modification in the name became necessary, so it was called the BUFFALO MEDICAL AND SURGICAL JOURNAL AND REPORTER. At this time the country was plunged in civil war and as a consequence there was deep commercial depression and distress, a period unfavorable for the commencement of such an enterprise. Hence it required no little courage and energy on the part of its editor to begin the reprinting of the JOURNAL at the time named; but the physicians of Buffalo had learned to appreciate the value of a good medical journal, all the more, perhaps, since they had been deprived of one.

The first number of the new series contained thirty-two pages, and the first volume an aggregate of 380 pages. With the beginning of the second volume the words "and Reporter" were dropped from its title and it was published under the name of the BUFFALO MEDI-

CAL AND SURGICAL JOURNAL until its semicentennial anniversary, August, 1895; then, with a view to simplicity, it dropped the words "and Surgical," and has since been known by its original name —THE BUFFALO MEDICAL JOURNAL.

For eighteen years Dr. Miner continued to publish the JOURNAL, though he was assisted a portion of the time by Dr. Edward N. Brush as associate editor, now superintendent of the Sheppard Asylum for the Insane, at Towson, Md. During the period of the war the pages of the JOURNAL became a historical record of the officers who entered the military service from Buffalo and vicinity. In the issue for June, 1869, may be found an account of the first application of the principles of enucleation, in the removal of ovarian and abdominal tumors, as performed by its originator, the editor, Dr. Julius F. Miner.

FRANK HAMILTON POTTER, M. D.

In 1879, Dr. Miner's failing health led him to resign his editorial work into other hands. The JOURNAL was sold to a syndicate of physicians composed of Thomas Lothrop, A. R. Davidson, Herman Mynter, Lucien Howe and P. W. Van Peyma. This administration began with Volume XIX., new series, August, 1879. The first volume published under the new management contained 556 pages, which indicated a steady growth in its size. With Volume XX., beginning August, 1882, the names of Drs. Howe and Mynter were dropped from the editorial staff, and two years later Dr. Van Peyma retired, leaving the JOURNAL in the hands of Drs. Lothrop and Davidson. The latter continued as managing editor until his death, May 25, 1888. In July, 1888, Dr. Davidson's interest in the magazine as well as his functions as managing editor passed into the hands of Dr. William Warren Potter, who has continued in their exercise since that time. In 1895 the jubilee number was published, giving a historical sketch of medical journalism and medical institutions from the establishment of the JOURNAL in 1845. At that time the JOUR-

NAL was enlarged to eighty pages and otherwise made to conform to the advancements of the age.

The three editors during its first series are dead; so, too, are Drs. A. R. Davidson, managing editor, F. R. Campbell and Frank Hamilton Potter, associate editors. Thus since the establishment of the JOURNAL, more than fifty years ago, six deaths have occurred in its editorial ranks. During the life-time of the JOURNAL nearly all the improvements in medicine and surgery that are valuable have been developed and it has served as a stimulus to continued effort on the part of the medical profession of Buffalo toward the advancement of medical science. It prides itself upon having kept pace with improvements, and so continued to display an energy worthy of professional esteem and support.

(*Continued next month.*)

## Figures and Tables

**Figure f1:**
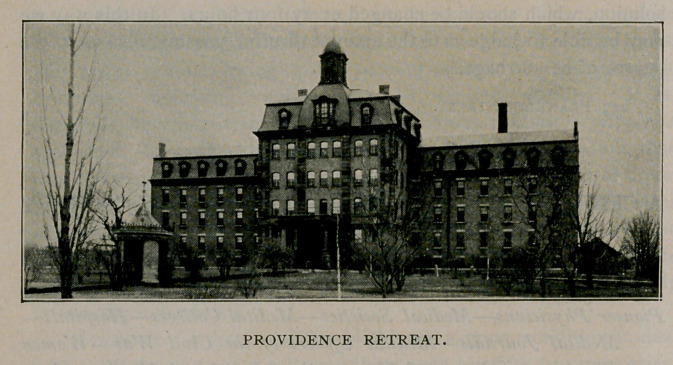


**Figure f2:**
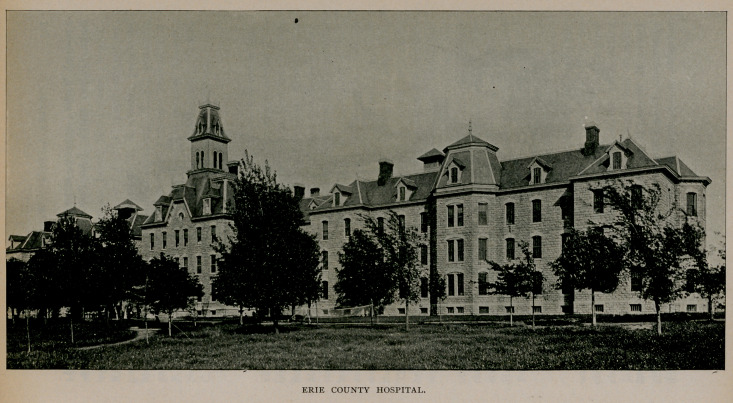


**Figure f3:**
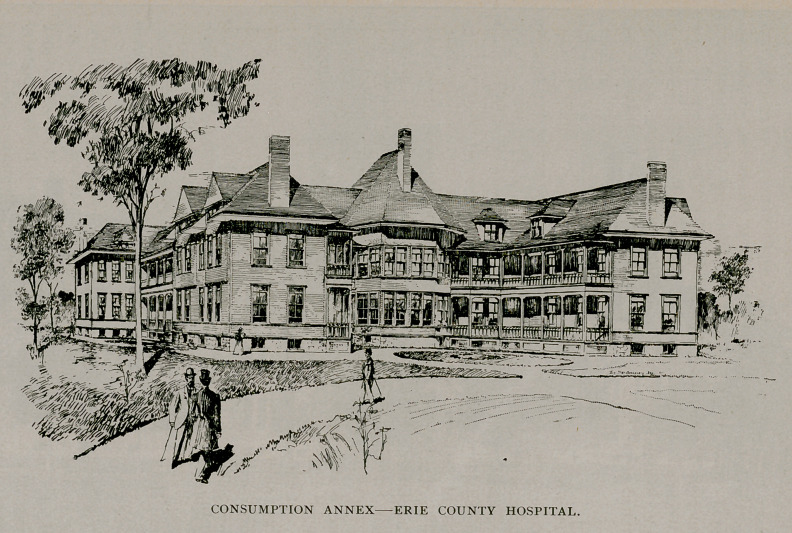


**Figure f4:**
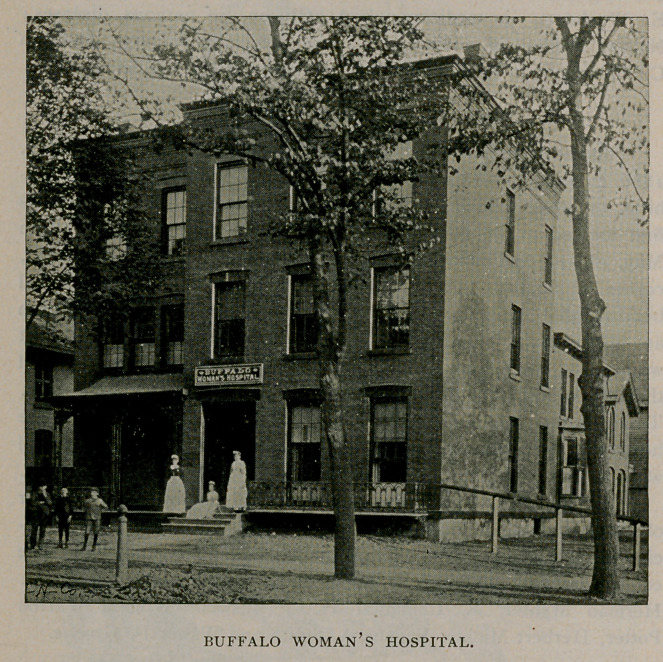


**Figure f5:**
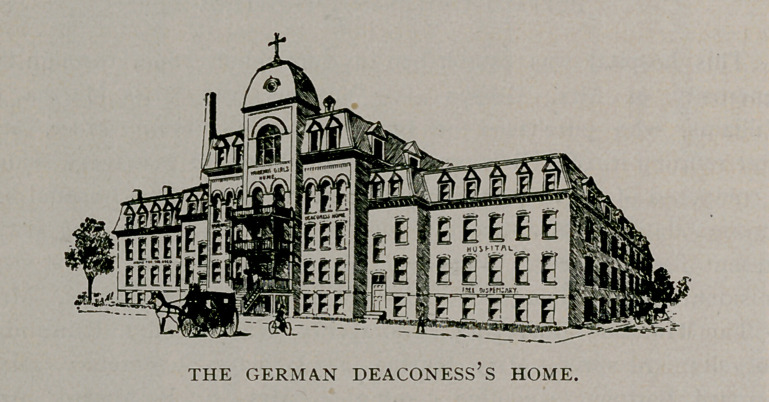


**Figure f6:**
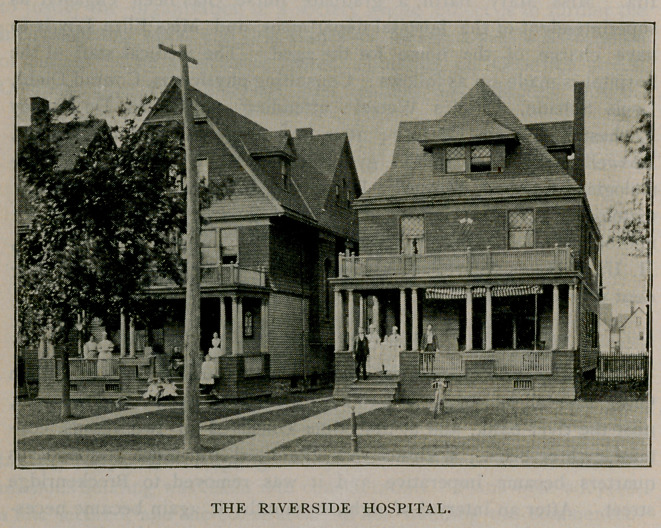


**Figure f7:**
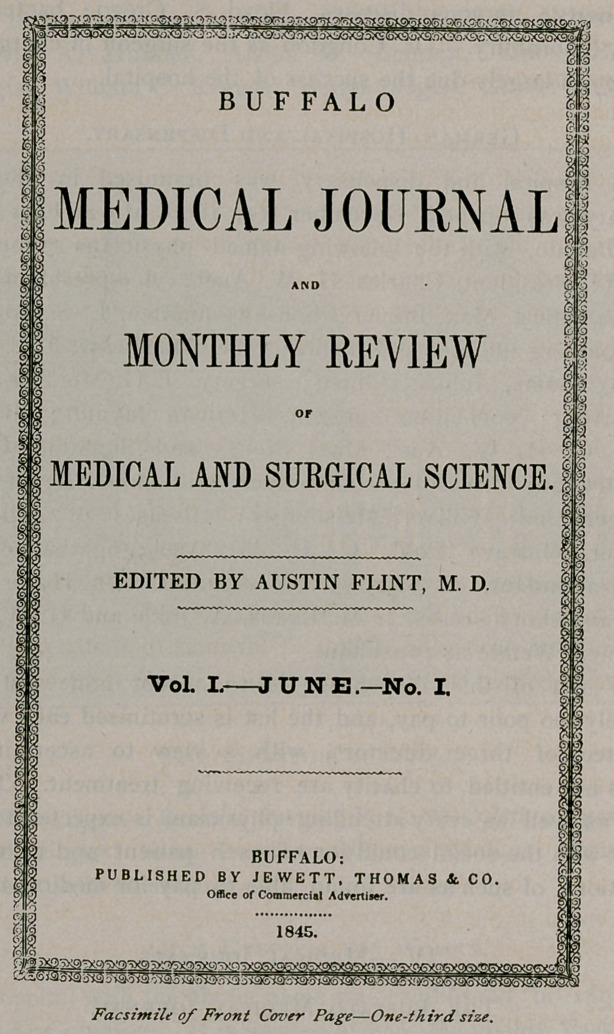


**Figure f8:**
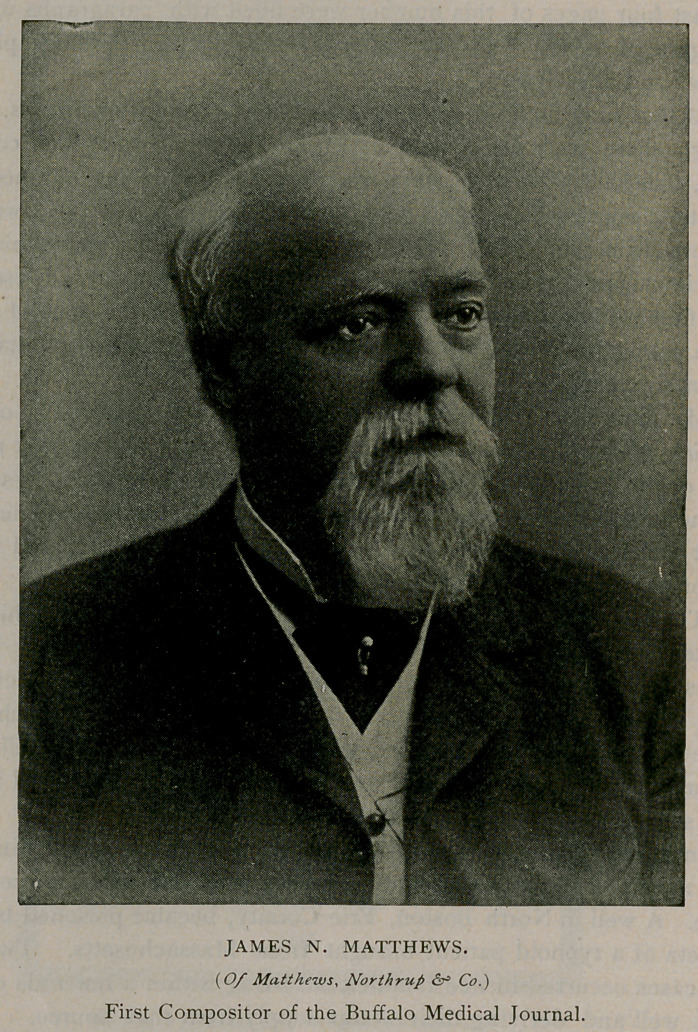


**Figure f9:**
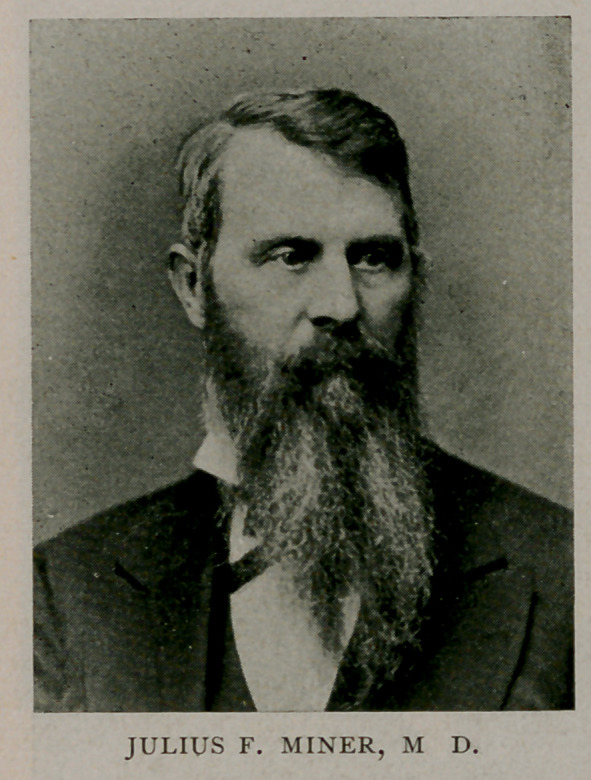


**Figure f10:**
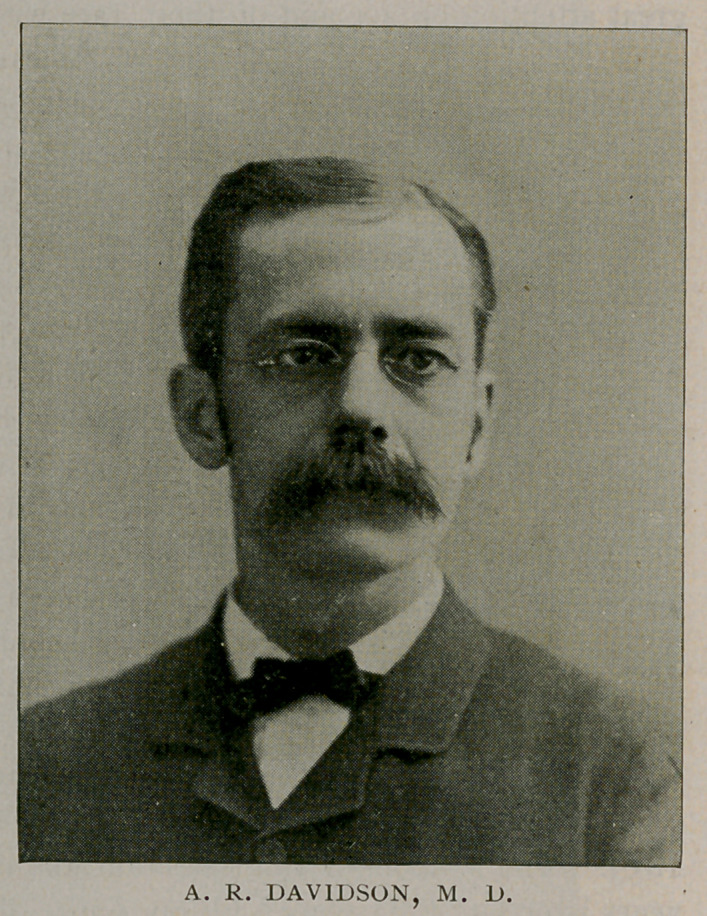


**Figure f11:**
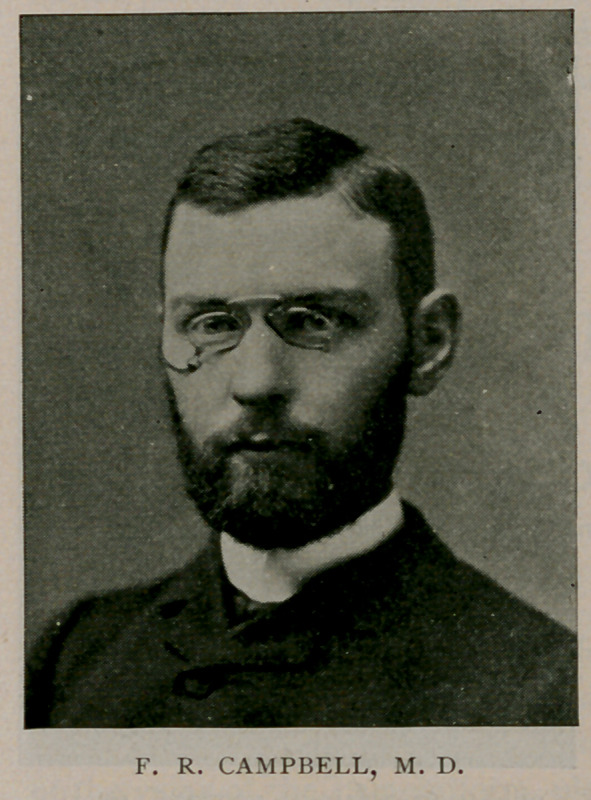


**Figure f12:**